# Supplementary material for: Stability of the standard incus coupling of the Carina middle ear actuator after 1.5T MRI
Source: PLoS One. 2020 Apr 9;15(4):e0231213. doi: 10.1371/journal.pone.0231213 (PMC7144975; doi:10.1371/journal.pone.0231213)
Supplement: Attachment — Submitted filename: Response to Reviewers.pdf [file pone.0231213.s002.pdf]

GUY FIERENS
DEPARTMENT OF NEUROSCIENCES, RESEARCH GROUP EXPORL
HERESTRAAT 49, BUS 721
3001 LEUVEN, BELGIUM
GUY.FIERENS@KULEUVEN.BE

Rafael da Costa Monsanto, M.D.
Academic Editor
PLOS ONE,
March 3, 2020

Revision of "Stability of the standard incus coupling of the Carina middle ear actuator after 1.5T MRI" manuscript (PONE-D-19-30029)

Dear Dr. da Costa Monsanto, dear reviewers,

We thank you for your time to review our manuscript and for the constructive feedback on our work. Based on the comments you provided, we have revised the manuscript. We agree that some aspects of the work had to be clarified to provide the PLOS ONE readers with a straightforward and unambiguous overview of our experiments. On the following pages of this letter we have addressed each reviewer's comment separately. Please share these replies with them on our behalf.

As the academic editor, familiar with the topic we investigated, you requested more information on how we have reached the conclusion that "the risk on dislocating the Carina middle ear actuator when coupled to the incus short process is limited." We have reached this conclusion based on the analysis of our CT image data and actuator impedance measurements. None of the measurement modalities have indicated that the actuator had become dislocated, leading us to the stated conclusion. We understand, however, that solely mentioning this sentence is unclear and have therefore clarified the conclusion section in the manuscript.

In addition, author affiliations have been updated to fit the PLOS ONE journal guidelines. Other formatting issues were not found when checking the journal requirements as we have used the available LaTex template for PLOS ONE submissions. The funding from Cochlear Ltd. has been mentioned in the comment section as we were not able to edit the competing interest statement directly. Finally, we have created a file containing all relevant data to allow replication of our study, which is added to the submission as supporting information. In case a researcher requires additional information, he/she can contact me directly as corresponding author.

On behalf of all contributing authors
Yours sincerely,

Guy Fierens

<u>Reviewer #1</u>: I read the manuscript entitled “Stability of the standard incus coupling of the Carina middle ear actuator after 1.5T MRI” with great interest. In this study, the authors aimed to measure the coupling stability of an actuator of a fully implantable AMEI after MRI scans. The subject of the study is clinically relevant, and the results are presented in an intelligible fashion. Although I commend the authors for tackling such an important subject, I do have a few concerns regarding the methodology that the authors could perhaps clarify.

<u>Answer</u>: We sincerely thank the reviewer for the positive feedback and the confidence in the clinical relevance of our work. We have updated the manuscript according to the comments below. Below we answered all questions point per point, indicating if and where we have made changes to the manuscript.

1. For stapedotomy, the angle between the prosthesis used and the incus long process is known to be ideal at 90 degrees (URL). I wonder that there must be perfect alignment between the actuator and the ossicular chain for optimal device performance. Why did the authors not perform these angle measurements?
   <u>Answer:</u> The optimal angle for coupling between the prosthesis and the incus long process is 90 degrees as indicated by the reviewer. Measuring and reporting on the angular values would optimize coupling efficiency and possibly also coupling stability. We have intentionally not performed angular measurements in order to avoid testing a “perfect situation”. We wanted to simulate a clinically relevant, yet not per se optimal solution to assess the risk of actuator dislocation in a slightly more worst-case. In addition, the coupling to the incus body, for sensorineural hearing loss indications, is different than a standard stapes surgery where the prosthesis needs to go into a stapedotomy hole.
2. The data from 3 TB CT scans was corrupted. Therefore, only 8 CT scans could be analyzed. I think this is a small sample to get further conclusions.
   <u>Answer:</u> We agree with the reviewer’s suggestion that a total of 8 samples is too little to draw statistically significant conclusions regarding any actuator displacement. However, our work focused at detecting any cases of actuator dislocation after MRI. To answer this research question we do believe that 8 samples is sufficient, especially considering the very consistent (and therefore relevant) results of the impedance analysis of all temporal bones. In a definite manner, the analysis of the CT data indicates that no movement greater than the pixel resolution of 0.3 mm occurred and that there are no clear indications of actuator dislocation in this data. The latter is then supported by the impedance analysis, which shows that the measured changes in impedance at the resonance frequency are well below the unloading threshold set by the manufacturer. We are therefore convinced that our data allows to answer our research question regarding the risk of actuator dislocation after an MRI exposure, despite the low number of available CT datasets.
3. What does LTA mean?
   <u>Answer:</u> The abbreviation TLA stands for “Transducer Loading Assistant”. This an older name for the Carina interface, which we forgot to update in the subtitle. Thank you for making us aware of this typo, it has been updated in the manuscript.
4. I believe that statistical analysis for mean impedance values (before and after MRI) are necessary.
   <u>Answer:</u> In order to detect a possible displacement, we compare the numerical value of the impedance difference before and after MRI with the manufacturer guidelines. These state that an impedance difference larger than 50 Ohm at the resonance frequency indicates actuator (de-)coupling to the ossicles. Making a statistical comparison between the impedance values themselves therefore does not add much value. To clarify why we have decided not to do a statistical analysis, we decided to include an additional figure in the manuscript that visualizes the difference between the obtained results and the (un-)loading threshold of 50 Ohm. For completeness and ease of reviewing we have pasted the image below this text. We hope this clarifies the reviewer’s concern.

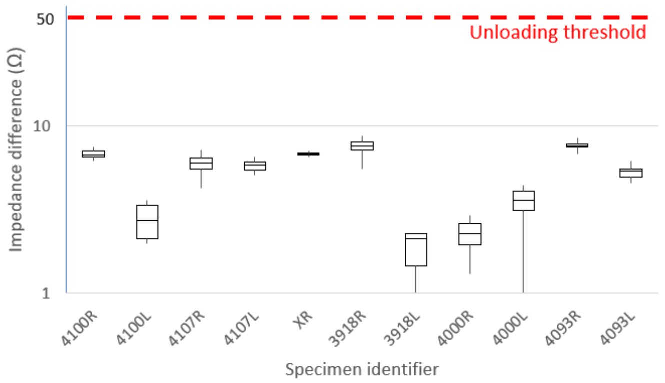
50
10
1
Unloading threshold
Impedance difference (Ω)
4100R
4100L
4107R
4107L
XR
3918R
3918L
4000R
4000L
4093R
4093L
Specimen identifier

<u>Reviewer #2</u>: Dear Author, your manuscript is well done and interesting. It is the first that evaluate the compatibility of the Carina Implant with the MRI. Your job is ready for the publication but one defect is that only the trasductor is studied. Can you evaluate all the device? You can stabilized implant and microphone with screws and then do the MRI. Please, let me know your opinion about this possibility.
<u>Answer</u>: We thank the author for the provided positive feedback and interesting question. With our work we can only make statements about the very specific aspect of MRI safety regarding actuator dislocation. Unfortunately, there are a number of other aspects that could affect the complete device during MRI, like: heating, unintended output, possible dislocation of the internal magnet, etc. More work is needed to evaluate all possible interactions before we can really make statements regarding the MRI safety of the entire Carina System. We are planning to tackle some of these aspects still within our group, but we encourage other groups to also look into the matter and support this topic of research. To clarify that we can not make conclusions regarding the complete implant's MRI safety, we have added a clarifying statement in the discussion section of the manuscript.

<u>Reviewer #3</u>: The authors of the present study conducted an extremely pertinent analysis of the subject in question. They clearly introduced the importance of magnetic resonance imaging in the growing world of implantable hearing aids. The study was conducted with fidelity of technical application, respecting the necessary steps to conduct the imaging exams in order to minimize the bias of selection or variation of radiological technique. The procedure of implantation of the anatomical specimens respected the indication criteria according to the latest manufacturer's guidelines. Actuator T2 dislocations were analyzed not only according to the computed tomography exam, which could lead us to a technical bias, but also according to the average of the impedance curves before and after 10 exams. MRI scan using the manufacturer's TLA. Finally, the authors concluded that and according to the results presented, little effect of the factor (magnetic resonance) on the implant analyzed, when using the classic anvil short coupling method.
<u>Answer:</u> We thank the reviewer for his supportive views on the presented work. We believe that MRI safety is an extremely important topic for patients with implanted devices. We hope that our work will contribute to the insights regarding the currently labelled MRI unsafe Carina. Even though we can not make statements regarding the full MRI safety of this device, the presented work already tackles a single aspect of the topic.
